# Effects of different cold-resistant agents and application methods on yield and cold-resistance of machine-transplanted early rice

**DOI:** 10.3389/fpls.2024.1422374

**Published:** 2024-10-02

**Authors:** Shuai Yuan, Shiqi Qin, Quan Shi, Pingping Chen, Naimei Tu, Wenxin Zhou, Zhenxie Yi

**Affiliations:** College of Agronomy, Hunan Agricultural University, Changsha, China

**Keywords:** rice yield, cold resistance characteristics, yield formation, application of cold-resistant agent, early rice

## Abstract

Cold stress is a critical factor affecting rice production worldwide. The application of cold-resistant agents may improve the cold resistance and yield of crops. To screen for suitable cold-resistant agents for machine-transplanted early rice, the effects of uniconazole, abscisic acid, and zinc-amino acids chelate and their spraying times (seed soaking stage, one leaf and one heart stage, two leaves and one heart stage, 7 days before the transplanting stage, and regreening stage) on the yield and cold resistance of machine-transplanted early rice were investigated. Moreover, the application method (spraying amount: 750 and 1125 g ha^−1^; spraying time: 7 days before the transplanting stage, transplanting stage, regreening stage, and transplanting stage and regreening stage) for the most suitable cold-resistant agent was optimized. The zinc-amino acids chelate was better than the other two cold-resistant agents for promoting rice tillering and increasing the leaf area index, dry matter weight, antioxidant enzyme activities (CAT, SOD, POD) and yield (i.e., 9.22% and 7.14% higher than uniconazole and abscisic acid, respectively), especially when it was applied in the regreening stage. The examination of spraying amounts and times indicated that the zinc-amino acids chelate dosage had no significant effect on the yield and cold resistance of early rice. However, the rice yield and antioxidant enzyme activities were highest when samples were sprayed once in the transplanting stage and the regreening stage. On the basis of the study results, 750 g ha^−1^ zinc-amino acids chelate applications in the transplanting and regreening stages of machine-transplanted early rice plants may be ideal for increasing cold stress resistance and yield.

## 1 Introduction

Rice is an important staple food crop consumed by a large proportion of the global population. Unfortunately, because of climate change, extreme weather conditions occur relatively frequently, with cold stress seriously affecting rice yield and quality (Huang et al., 2013). As one of the main grain-producing areas in China, the southern rice-growing region is susceptible to late spring cold conditions, which are mainly caused by long periods of rainfall, frequent intrusion of cold air, or radiative cooling due to cold anticyclones on clear nights (Qian and Zhang, 2012). Cold stress seriously affects the production of early rice (e.g., decreased seedling quality as well as delayed transplanting, regreening of tillers, and panicle differentiation) and may even affect the planting time of late rice (Ai et al., 2022; Crofts et al., 2022; Kwak et al., 2018; Zhou et al., 2018). Extreme low-temperature stress in Hunan province from 1981 to 2010 reportedly decreased early rice and late rice yields by 6.66% and 1.82%, respectively (Liu et al., 2019).

Mechanized rice planting is currently the simplest and most effective cultivation method for rice at least partly because it can decrease the required labor force and optimize the economic benefits of rice production (Li et al., 2022). Machine transplanting has significantly increased rice production efficiency by decreasing costs, time, and labor, thereby increasing productivity and profits. However, the problems associated with this approach include low seedling quality and limited seedling age elasticity, which seriously restricts the utility of machine-transplanted rice seedlings (Gao et al., 2022). In addition, early rice seedlings are susceptible to low-temperature stress in southern China, further limiting the development and application of machine-transplanting technology.

Cold-induced damages significantly affect rice plant growth, development, and physiological metabolism. Earlier research showed that rice plants are most susceptible to low-temperature stress at the seedling and panicle differentiation stages. An exposure to low temperatures will decrease the seedling establishment rate and lead to panicle degeneration, resulting in a significant decrease in rice yield (Zhang et al., 2022). Low temperatures can also directly affect antioxidant enzyme activities, chlorophyll contents, and photosynthetic rates in early rice leaves. Specifically, low-temperature stress during the rice seedling stage decreases superoxide dismutase (SOD) and peroxidase (POD) activities in leaves, while increasing the malondialdehyde content (Theocharis et al., 2012) and decreasing the photosynthetic capacity (Zsiros et al., 2019). Cold-induced damages to rice plants may be restricted by certain defensive measures, including the selection of cold-resistant varieties (Lee and Kwon, 2015), warm water irrigation (Zhang et al., 2020), or soil warming (Ge et al., 2015). However, there are potential issues and problems associated with these measures (e.g., varietal differences among rice-growing regions, high management costs, and detrimental effects on ecological systems). Foliar fertilizers are widely used to increase crop cold resistance and yield because of their low cost and the fact they are not particularly harmful to the environment. Gurav et al. (2019) determined that potassium dihydrogen phosphate can protect plant seedlings from low-temperature stress because it stabilizes the plant cell membrane structure to maintain its protective function and permeability. Li et al. (2015) observed that potassium and zinc in foliar fertilizers can decrease oxidative damage due to reactive oxygen species in plants under low temperature stress, and improve the growth and development of plants. Pretreatment of rice seedlings with SA (salicylic acid) induced enhanced cold tolerance, mainly manifested in increased antioxidant system activity, thereby improving the relationship between yield components and increasing grain yield (Huang et al., 2016; Wang et al., 2021a).

The effects of low temperatures on rice yield formation and physiological characteristics have been extensively studied (Crofts et al., 2022; Jia et al., 2022; Xu et al., 2020). Research on technical measures that affect low-temperature defense responses has mostly focused on cold-induced damages and the expression of cold tolerance-related genes during the seedling stage (Wang et al., 2021b; Yang et al., 2012). Relatively few studies have screened different types of cold-resistant agents useful for treating machine-transplanted early rice in subtropical regions. Furthermore, there are no reports on the effects of their application methods on the cold-resistance of rice. In the first year of this study, we examined the effects of different cold-resistant agents and their spraying times on the early rice yield and seedling cold resistance under machine-transplanted conditions to identify the most suitable cold-resistant agent. In the second year, we analyzed the techniques used to apply a selected cold-resistant agent to determine the optimal spraying amount and time for early rice. By identifying appropriate cold-resistant agents and application methods, we provide theoretical and technical support for improving the cultivation of stress-resistant and stable-yielding rice plants.

## 2 Materials and methods

### 2.1 Test site and materials

This study was conducted at the Field Experiment Station of the Agriculture and Rural Affairs Bureau of Hengyang county, Hunan province, China (26° 97′ N, 111° 37′ E) in 2018 and 2019. The soil nutrient conditions were as follows: 1.46 g kg^-1^ total N, 0.61 g kg^-1^ total phosphorus, 18.87 g kg^-1^ total potassium, 158.44 mg kg^-1^ alkaline hydrolysis N, 8.93 mg kg^-1^ available phosphorus, 114.07 mg kg^-1^ available potassium, and 26.01 g kg^-1^ organic matter in 2018, and correspondingly 1.53 g kg^-1^, 0.57 g kg^-1^, 19.05 g kg^-1^, 169.12 mg kg^-1^, 9.73 mg kg^-1^, 109.95 mg kg^-1^ and 25.68 g kg^-1^ in 2019. Meteorological data of daily average temperature in Hengyang County, Hengyang City, Hunan Province, China were collected from March to July in 2018 and 2019 (**Supplementary Figure S1**). The data comes from the National Meteorological Science Data Center (http://data.cma.cn). For some missing data, the average value of the meteorological element in the adjacent 2 days on that day is used as a replacement.

Zhongjiazao 17, which is an *indica* type conventional early rice variety with a growth period of 109 days, was used in both years. The following cold-resistant agents were analyzed: uniconazole aqueous agent (180 mg L^−1^) produced by Chongqing Tuyouta Company (China), abscisic acid aqueous agent (15 mg L^−1^) produced by Henan Zhifu Company (China), and zinc-amino acids chelate (ZAC; i.e., compound foliar fertilizer) powder (amino acids ≥100 g kg^−1^; Zn ≥50 g kg^−1^) produced by Hunan Guonong Company (China).

### 2.2 Experimental design

A two-factor experiment involving different cold-resistant agents and spraying times was conducted in 2018. The cold-resistant agents were tested in the main area, whereas the spraying times were tested in the secondary area. The cold-resistant agents were uniconazole (C1: 75 ml m^−2^), abscisic acid (C2: 75 ml m^−2^), and ZAC (C3: 650 g ha^−1^). The spraying times included the seed soaking stage (D1), one leaf and one heart stage (D2), two leaves and one heart stage (D3), 7 days before the transplanting stage (D4), and regreening stage (D5; 7 days after the transplanting stage). Fifteen treatments were included in the experiment, with each treatment conducted three times for a total of 45 plots.

In 2019, a two-factor experiment with different spraying amounts and spraying times was conducted using ZAC. The spraying amounts were tested in the main area, whereas the spraying times were tested in the secondary area. The spraying amounts were 750 g ha^−1^ (B1) and 1125 g ha^−1^ (B2). The spraying times were 7 days before the transplanting stage (A1), transplanting stage (A2), regreening stage (A3), and transplanting stage and regreening stage (A4). For each spraying amount, plants were sprayed twice. A control treatment (CK) group was also included. The experiment comprised nine treatments, with each treatment conducted three times for a total of 27 plots.

In both years, early rice seeds were soaked on March 24 and sown on March 28, with the resulting seedlings transplanted on April 20. The size of the test seedling tray was 58 cm × 28 cm. The machine planting density was 30 cm × 11 cm. The study field area was 20 m^2^, and the surrounding area was separated by protective rows covered with polyethylene plastic films (0.3 m width and 0.3 m height). Independent irrigation and drainage outlets were provided. Other management measures were in accordance with local practices.

### 2.3 Measurement items and methods

Starting on day 5 after transplanting, 20 consecutive and representative rice plants with consistent growth were selected in each plot. The number of rice tillers was recorded every 5 days. To examine the leaf area and dry weight during critical growth stages (tillering, booting, heading, milky, and maturity), three plants were sampled from each plot. The leaf area was measured using a length–width coefficient method (Park et al., 2004). The plant samples at each stage were divided into the leaf, stem, and panicle and bagged, cured at 105°C for 30 min, and dried to a constant weight at 80°C. The dry matter weight of each plant part was recorded.

At maturity, 80 rice plants in each plot were examined to calculate the effective panicle number per plant. On the basis of the average effective panicle number, five plants per plot were sampled and examined in the laboratory to determine the number of grains per panicle, seed setting rate, and thousand-grain weight. Next, in each plot, 80 randomly selected rice plants were harvested, but not from the outer three rows. After threshing, the straw and empty grains were removed before grains were weighed and the moisture content was measured according to the drying method. The actual yield was calculated using a moisture content of 13.5%. The following formula was used: actual output = harvested output × (1 − moisture content)/0.865.

In 2018, rice leaves were collected at 7 days after the regreening stage, whereas in 2019, rice leaves were collected at the 7 days after the regreening stage, peak tillering stage, booting stage, and milky stage. Extract the test solution according to the method of Xie et al. (2008). Weigh a small amount of fresh rice leaves, cut them into pieces and put them in a mortar. Add liquid nitrogen to cover the leaves, let it stand for a few minutes and then quickly grind it into powder. Then use the One ten thousandth of the balance scale(AB135-S, Mettler Toledo, Zurich, Switzerland) to weigh 0.1 grams (± 0.0005g) of the sample and put it in a centrifuge tube. Add 1.5 ml of phosphate buffer (pH 7.8) and centrifuge at 10,000 r·min^-1^ at 4°C for 20 min. Then transfer the supernatant to a 20 ml graduated test tube and dilute to 15 ml with 62.5 mmol·L^-1^ phosphate buffer and mix well for later use. Repeat each treatment 3 times. The enzyme activities of CAT, SOD, and POD were determined according to the method of Munir et al. (2021). That is, a UV-visible spectrophotometer (UV2700, Shimadzu, Kyoto, Japan) was used to measure the absorbance values of the corresponding indicators (SOD, POD, and CAT) of the extracted enzyme solution at different wavelengths, and then the enzyme activity was calculated.

### 2.4 Statistical analysis

One-way analysis of variance (ANOVA) was used for Duncan’s multiple comparisons (P<0.05) of different treatments using SPSS 24 for Windows (IBM, Armonk, NY, USA). Graphs were plotted by Origin 2023 (OriginLab, Northampton, MA, USA).

## 3 Results

### 3.1 Screening of suitable cold-resistant agents for machine-transplanted early rice

#### 3.1.1 Rice tillering dynamics

**Figure 1:**
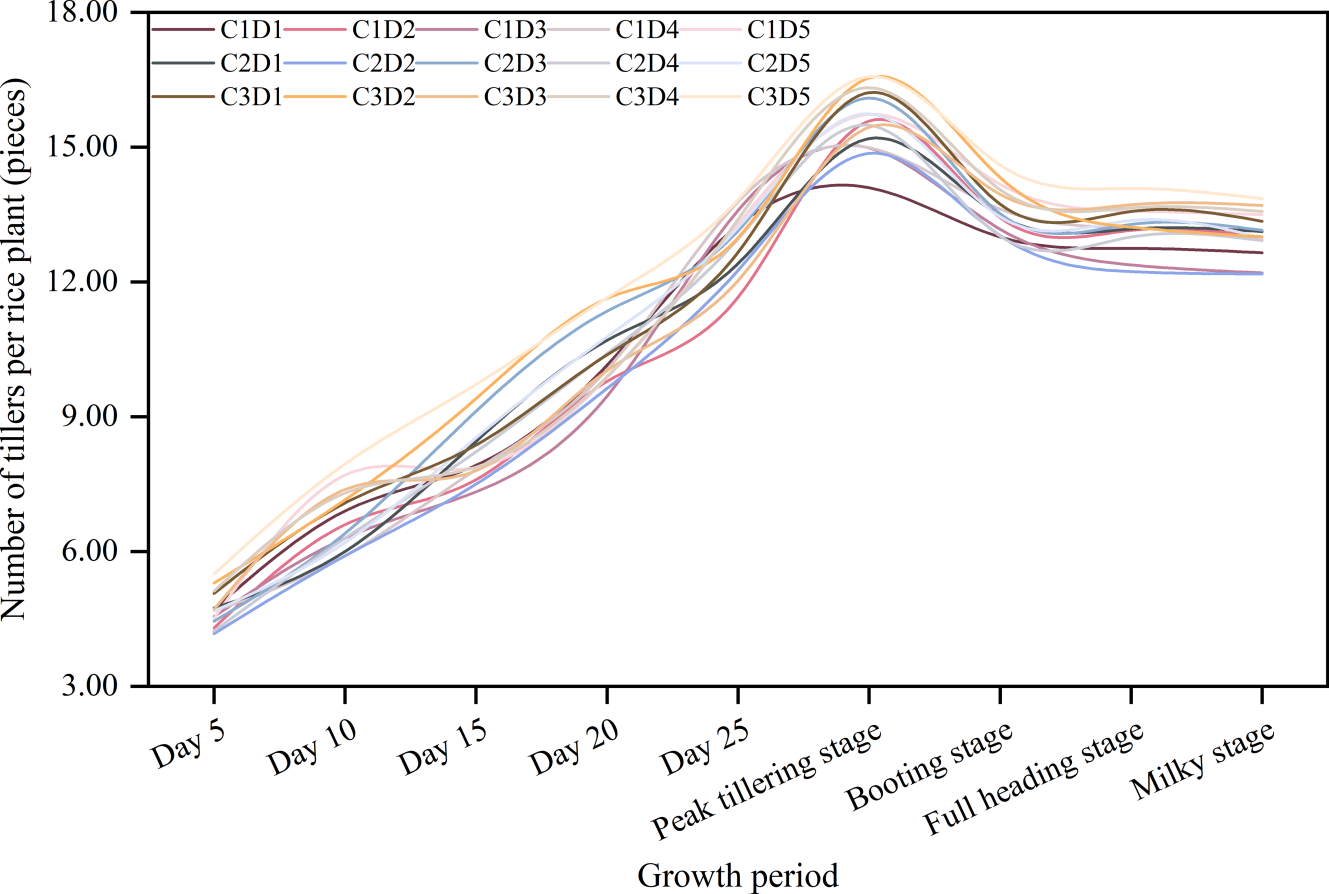
Effects of different cold-resistant agents and their spraying times on rice tillering dynamics. C1: Uniconazole; C2: Abscisic acid; C3: Zinc-amino acids chelate; D1: Seed soaking stage; D2: One leaf and one heart stage; D3: Two leaves and one heart stage; D4: 7 days before the transplanting stage; D5: regreening stage.

The number of tillers initially increased up to the peak tillering stage and then decreased (**Figure 1**). In terms of the effects of cold-resistant agents, the number of tillers per plant was highest for C3 at each growth stage. For C3, C2, and C1, the number of tillers per plant in the peak tillering stage was 16.22, 15.47, and 15.08, respectively. There were no major differences in the number of tillers at each growth stage among the spraying times. In the peak tillering stage, the number of tillers was highest for D5 and D2 (16.01 and 15.66 tillers per plant, respectively), followed by D4 and D3 (15.60 and 15.50 tillers per plant, respectively). The D1 treatment resulted in the fewest tillers (15.17 tillers per plant). Among the combined treatments, the number of tillers at each growth stage was highest for C3D5 (16.57 tillers per plant). Considered together, these results suggest an ZAC treatment during the regreening stage may be ideal for maximizing the number of tillers.

#### 3.1.2 Dry matter weight

The comparison of the effects of cold-resistant agents revealed the dry matter weight at each growth stage was highest for C3, followed by C2 and then C1 (**Table 1**). More specifically, the dry matter weight was significantly higher for C3 than for the other two treatments from the tillering stage to the full heading stage. Moreover, there was a significant difference in the dry matter weight between C3 and C1 in the milky stage and maturity stage. In terms of the spraying times, with the exception of the tillering stage, the dry matter weight was highest for D5, followed by D2. The differences in the dry matter weight between D5 and the other treatments were significant. For the combined treatments, C3D1 resulted in the highest dry matter weight in the tillering stage, whereas C3D5 produced the highest dry matter weight from the booting stage to the maturity stage.

**Table 1:** Effects of different cold-resistant agents and their spraying times on the early rice dry matter weight (g plant^-1^).

Treatment		Peak tillering stage	Booting stage	Full-heading stage	Milky stage	Maturity stage
Cold-resistant	C1	5.15 ± 0.52c	16.18 ± 0.44b	24.99 ± 0.64b	33.38 ± 0.94b	40.59 ± 1.25b
	C2	5.50 ± 0.38b	16.35 ± 0.53b	25.84 ± 0.50b	35.37 ± 1.37ab	42.56 ± 1.17ab
	C3	5.90 ± 0.69a	17.42 ± 0.48a	27.62 ± 1.13a	37.32 ± 1.22a	43.87 ± 1.40a
Spraying time	D1	5.77 ± 0.34a	15.95 ± 0.75b	25.62 ± 0.65b	34.70 ± 0.93b	39.72 ± 1.26b
	D2	5.11 ± 0.21b	17.76 ± 0.66a	26.76 ± 0.71ab	36.09 ± 0.97ab	44.81 ± 1.57a
	D3	5.75 ± 0.37a	15.43 ± 0.73b	26.60 ± 0.57ab	34.42 ± 1.21b	40.53 ± 1.18b
	D4	5.45 ± 0.29ab	15.76 ± 0.52b	24.57 ± 0.40c	33.94 ± 1.14b	40.74 ± 1.09b
	D5	5.60 ± 0.26a	18.36 ± 0.91a	27.21 ± 0.83a	38.12 ± 1.05a	45.06 ± 1.49a
Cold-resistant× Spraying time	C1D1	5.40 ± 0.18cd	16.00 ± 0.51cd	24.32 ± 0.38de	32.04 ± 0.95e	33.66 ± 0.91f
	C1D2	4.32 ± 0.17f	18.65 ± 0.77a	25.42 ± 0.59cd	30.06 ± 0.62f	43.96 ± 1.13bc
	C1D3	5.75 ± 0.25c	13.81 ± 0.48e	26.61 ± 0.51bc	34.24 ± 0.83cd	42.78 ± 1.03bc
	C1D4	5.02 ± 0.15e	13.83 ± 0.32e	23.87 ± 0.55e	35.77 ± 1.07bc	39.27 ± 0.89e
	C1D5	5.36 ± 0.21d	18.20 ± 0.65ab	24.75 ± 0.67de	34.79 ± 1.09bcd	42.91 ± 1.07bc
	C2D1	4.31 ± 0.12f	16.76 ± 0.42bc	24.89 ± 0.31d	36.35 ± 1.14b	39.03 ± 0.94e
	C2D2	5.45 ± 0.17cd	15.83 ± 0.38d	26.53 ± 0.47bc	35.90 ± 1.03bc	44.19 ± 1.18b
	C2D3	5.70 ± 0.22c	15.15 ± 0.35d	25.76 ± 0.44cd	33.55 ± 0.89de	40.95 ± 0.81d
	C2D4	6.25 ± 0.31b	15.68 ± 0.40d	23.80 ± 0.56e	30.56 ± 0.47f	40.94 ± 0.75d
	C2D5	5.92 ± 0.27bc	18.35 ± 0.83ab	28.03 ± 0.91a	35.87 ± 1.31bc	46.19 ± 1.14a
	C3D1	7.61 ± 0.43a	15.09 ± 0.51d	27.65 ± 0.65ab	35.02 ± 1.24bcd	45.67 ± 1.03ab
	C3D2	5.57 ± 0.19c	18.41 ± 0.61a	28.22 ± 0.88a	43.01 ± 2.37a	46.52 ± 1.37a
	C3D3	5.80 ± 0.20bc	17.35 ± 0.39b	27.63 ± 0.73ab	35.46 ± 1.12bc	37.85 ± 0.99e
	C3D4	5.10 ± 0.10de	17.75 ± 0.52ab	26.03 ± 0.61c	35.81 ± 1.18bc	42.01 ± 0.93cd
	C3D5	5.54 ± 0.14cd	18.91 ± 0.82a	28.77 ± 1.06a	43.40 ± 3.02a	46.60 ± 1.29a

C1: Uniconazole; C2: Abscisic acid; C3: Zinc-amino acids chelate; D1: Seed soaking stage; D2: One leaf and one heart stage; D3: Two leaves and one heart stage; D4: 7 days before the transplanting stage; D5: regreening stage. For each treatment (C, D, or C × D), different letters in the same column indicate significant differences (P < 0.05).

#### 3.1.3 Leaf area index

The early rice leaf area index (LAI) was significantly higher for C3 than for the other two treatments in each growth stage (**Table 2**). The comparison of the spraying times indicated there were no significant differences in LAI among treatments in the tillering stage, but LAI was highest and lowest for D5 and D4, respectively, from the booting stage to the milky stage; this difference was significant. For the combined treatments, C3D1 resulted in the highest LAI in the tillering stage, but C3D5 produced the highest LAI from the booting stage to the milky stage. Accordingly, the early rice LAI was highest following the application of ZAC in the regreening stage.

**Table 2:** Effects of different cold-resistant agents and their spraying times on the early rice LAI.

Treatment		Peak tillering stage	Booting stage	Full-heading stage	Milky stage
Cold-resistant	C1	2.09 ± 0.17b	5.56 ± 0.15b	4.54 ± 0.18b	3.82 ± 0.22b
	C2	2.24 ± 0.20b	5.62 ± 0.18b	4.74 ± 0.15b	3.76 ± 0.26b
	C3	2.76 ± 0.29a	6.13 ± 0.22a	5.27 ± 0.23a	4.47 ± 0.31a
Spraying time	D1	2.42 ± 0.22a	5.63 ± 0.28bc	5.10 ± 0.23a	3.81 ± 0.16c
	D2	2.49 ± 0.19a	5.53 ± 0.33bc	4.83 ± 0.19ab	4.26 ± 0.19ab
	D3	2.60 ± 0.27a	5.79 ± 0.25b	4.98 ± 0.20a	4.17 ± 0.11b
	D4	2.35 ± 0.21a	5.26 ± 0.19c	4.60 ± 0.16b	3.77 ± 0.20c
	D5	2.25 ± 0.24a	6.55 ± 0.41a	5.04 ± 0.25a	4.46 ± 0.17a
Cold-resistant × Spraying time	C1D1	2.34 ± 0.19de	6.04 ± 0.14b	4.83 ± 0.20cd	3.46 ± 0.14d
	C1D2	1.91 ± 0.15e	4.97 ± 0.13e	4.73 ± 0.11de	3.81 ± 0.19c
	C1D3	2.27 ± 0.23de	5.32 ± 0.20d	4.68 ± 0.15de	4.22 ± 0.18b
	C1D4	2.04 ± 0.14e	5.39 ± 0.11d	4.26 ± 0.17f	3.72 ± 0.12c
	C1D5	2.10 ± 0.20e	6.10 ± 0.15b	4.47 ± 0.18ef	4.30 ± 0.15ab
	C2D1	1.64 ± 0.11f	5.27 ± 0.15d	4.42 ± 0.13ef	3.51 ± 0.17d
	C2D2	2.78 ± 0.21bc	5.28 ± 0.15d	4.80 ± 0.22d	4.33 ± 0.20ab
	C2D3	2.51 ± 0.21cd	5.57 ± 0.12cd	5.10 ± 0.16bc	3.71 ± 0.25c
	C2D4	2.47 ± 0.17cd	5.50 ± 0.17cd	4.48 ± 0.12ef	3.54 ± 0.19c
	C2D5	2.10 ± 0.22e	6.48 ± 0.21a	5.22 ± 0.17b	4.10 ± 0.14b
	C3D1	3.28 ± 0.26a	5.57 ± 0.22cd	5.25 ± 0.19b	4.46 ± 0.19a
	C3D2	2.68 ± 0.15cd	6.49 ± 0.19a	5.55 ± 0.25ab	4.64 ± 0.24a
	C3D3	3.03 ± 0.16ab	5.85 ± 0.26bc	5.18 ± 0.15b	4.57 ± 0.17a
	C3D4	2.56 ± 0.26cd	6.08 ± 0.17b	5.06 ± 0.18bc	4.06 ± 0.22b
	C3D5	2.55 ± 0.24cd	6.65 ± 0.29a	5.72 ± 0.26a	4.89 ± 0.23a

C1: Uniconazole; C2: Abscisic acid; C3: ZincAmino Acids Chelate; D1: Seed soaking stage; D2: One leaf and one heart stage; D3: Two leaves and one heart stage; D4: 7 days before the transplanting stage; D5: regreening stage. For each treatment (C, D, or C × D), different letters in the same column indicate significant differences (P < 0.05).

#### 3.1.4 Yield and related components

The rice yield was significantly higher for C3 than for C1 and C2 (i.e., 9.22% and 7.14% higher, respectively; **Table 3**). In terms of the yield-related components, the number of grains per panicle and seed setting rate were significantly higher for C3 than for C1 and C2. For the spraying time treatments, D5 produced the highest rice yield, followed by D2 and D1. The rice yield was lowest for D3. Notably, the rice yields for D2 and D5 were significantly higher than those for D1 and D3. In terms of the yield-related components, there were no significant differences in the effective panicle number and thousand-grain weight between treatments. The number of grains per panicle was significantly higher for D2, D4, and D5 than for D1 and D3. The seed setting rate was significantly lower for D3 than for the other treatments. Among the combined treatments, C3D5 and C1D1 resulted in the highest and lowest early rice yields, respectively. The number of grains per panicle and seed setting rate were significantly higher for C3D5 than for the other treatments, except for C3D2.

**Table 3:** Effects of different cold-resistant agents and their spraying times on the early rice yield and related components.

Treatment		Effective panicle (×10^4^ ha^-1^)	Grains per panicle	Seed setting rate (%)	Thousand -grain weight (g)	Theoretical Yield (t ha^-1^)	Actual yield (t ha^-1^)
Cold-resistant	C1	251.84 ± 9.22a	109.16 ± 1.87b	66.52 ± 2.04b	24.06 ± 1.67a	4.40 ± 0.21b	4.12 ± 0.15b
	C2	263.36 ± 11.76a	107.02 ± 2.05b	64.84 ± 2.27b	24.15 ± 2.33a	4.43 ± 0.19b	4.20 ± 0.11b
	C3	250.84 ± 7.31a	113.46 ± 2.27a	70.93 ± 2.35a	24.17 ± 1.73a	4.89 ± 0.24a	4.50 ± 0.17a
Spraying time	D1	252.10 ± 8.65a	104.80 ± 2.46b	68.12 ± 1.87a	24.22 ± 1.93a	4.36 ± 0.17b	4.07 ± 0.12b
	D2	254.60 ± 10.09a	111.83 ± 2.86a	67.47 ± 1.29a	24.06 ± 2.08a	4.78 ± 0.21a	4.41 ± 0.21a
	D3	259.50 ± 10.59a	107.13 ± 2.05b	64.88 ± 1.15b	24.16 ± 2.31a	4.37 ± 0.20b	4.07 ± 0.10b
	D4	249.60 ± 7.91a	113.13 ± 3.38a	67.71 ± 1.44a	24.20 ± 1.87a	4.63 ± 0.24ab	4.35 ± 0.17ab
	D5	260.93 ± 11.48a	112.50 ± 3.10a	68.97 ± 2.21a	23.99 ± 2.15a	4.85 ± 0.27a	4.51 ± 0.26a
Cold-resistant× Spraying time	C1D1	231.80 ± 11.24c	106.20 ± 4.31cd	62.09 ± 3.17def	24.38 ± 1.68a	3.73 ± 0.13f	3.42 ± 0.27e
	C1D2	266.43 ± 10.93ab	111.48 ± 3.66bc	70.62 ± 4.75bc	23.75 ± 2.04a	4.97 ± 0.22b	4.81 ± 0.19b
	C1D3	254.48 ± 7.15bc	115.58 ± 3.04b	66.83 ± 4.07cde	24.10 ± 1.47a	4.73 ± 0.20bc	4.50 ± 0.15bc
	C1D4	232.40 ± 7.09c	111.34 ± 5.84bc	64.10 ± 2.89de	23.33 ± 3.08a	4.03 ± 0.17e	3.73 ± 0.17de
	C1D5	275.19 ± 12.16a	101.40 ± 5.04de	68.95 ± 2.24cd	23.75 ± 2.59a	4.57 ± 0.11c	4.18 ± 0.16d
	C2D1	266.46 ± 11.57ab	111.96 ± 4.61bc	69.62 ± 3.46bc	23.63 ± 1.61a	4.90 ± 0.30b	4.73 ± 0.20b
	C2D2	243.28 ± 10.54c	101.57 ± 4.49de	57.10 ± 3.12f	24.08 ± 1.38a	3.40 ± 0.14g	3.19 ± 0.10f
	C2D3	272.65 ± 9.05a	104.56 ± 6.10cde	66.25 ± 3.81cde	23.54 ± 1.95a	4.62 ± 0.18c	4.31 ± 0.11c
	C2D4	272.13 ± 10.27a	106.20 ± 5.04cd	70.71 ± 2.59bc	24.41 ± 2.23a	4.99 ± 0.25b	4.75 ± 0.17b
	C2D5	263.77 ± 7.35ab	111.05 ± 4.65bc	60.52 ± 4.09ef	24.10 ± 3.20a	4.26 ± 0.24de	4.05 ± 0.14d
	C3D1	258.50 ± 8.17abc	96.36 ± 2.81e	72.66 ± 4.20abc	24.65 ± 2.81a	4.46 ± 0.23cd	3.96 ± 0.18d
	C3D2	254.27 ± 9.68bc	122.69 ± 4.28a	74.69 ± 4.14ab	24.36 ± 3.05a	5.67 ± 0.38a	5.22 ± 0.21a
	C3D3	252.53 ± 11.31bc	101.40 ± 5.04de	61.57 ± 2.76ef	23.84 ± 1.43a	3.76 ± 0.10f	3.49 ± 0.20e
	C3D4	244.41 ± 9.25c	121.93 ± 3.35a	68.31 ± 2.42cd	23.86 ± 1.96a	4.86 ± 0.21b	4.57 ± 0.14b
	C3D5	244.83 ± 10.40c	125.10 ± 6.44a	77.44 ± 3.60a	24.13 ± 3.21a	5.72 ± 0.32a	5.29 ± 0.27a

C1: Uniconazole; C2: Abscisic acid; C3: Zinc-amino acids chelate; D1: Seed soaking stage; D2: One leaf and one heart stage; D3: Two leaves and one heart stage; D4: 7 days before the transplanting stage; D5: regreening stage. For each treatment (C, D, or C × D), different letters in the same column indicate significant differences (P < 0.05).

#### 3.1.5 Rice leaf cold resistance characteristics

The CAT, SOD, and POD activities were highest for C3, followed by C2 and then C1, with significant differences between treatments (**Table 4**). Additionally, the CAT, SOD, and POD activities were highest for D5, followed by D2. The activities of these three enzymes were significantly lower for the other treatments. For the combined treatments, the CAT, SOD, and POD activities were highest and lowest for C3D5 and C1D3, respectively, with significant differences among treatments. Thus, the antioxidant capacity of leaves peaked when ZAC was applied at the regreening stage.

**Table 4:** Effects of different cold-resistant agents and their spraying times on the early rice leaf antioxidant system.

Treatment		CAT activity [U·(g·min)^-1^]	SOD activity (U g^-1^)	POD activity [U·(g·min)^-1^]
Cold-resistant	C1	103.25 ± 5.97c	105.19 ± 5.01c	123.64 ± 7.46c
	C2	115.68 ± 4.57b	146.10 ± 5.74b	156.17 ± 8.09b
	C3	128.49 ± 7.13a	158.54 ± 7.51a	181.41 ± 11.17a
Spraying time	D1	103.37 ± 6.70c	116.10 ± 4.03c	143.19 ± 8.22b
	D2	129.54 ± 4.94a	157.68 ± 10.26a	160.59 ± 5.21a
	D3	93.66 ± 3.72d	123.50 ± 3.15b	138.83 ± 9.07b
	D4	119.59 ± 5.65b	125.69 ± 5.28b	146.26 ± 8.39b
	D5	132.91 ± 7.28a	160.06 ± 8.17a	179.84 ± 10.11a
Cold-resistant× Spraying time	C1D1	90.79 ± 4.68g	98.67 ± 5.27f	112.40 ± 5.57f
	C1D2	119.19 ± 4.66d	117.02 ± 9.12e	129.13 ± 9.03e
	C1D3	87.89 ± 5.65g	93.24 ± 6.08f	106.03 ± 7.13f
	C1D4	107.19 ± 7.35ef	95.87 ± 4.16f	115.88 ± 4.21f
	C1D5	111.19 ± 4.46def	121.14 ± 7.37e	154.78 ± 8.72d
	C2D1	107.29 ± 5.65ef	119.19 ± 8.41e	138.78 ± 7.83e
	C2D2	128.90 ± 6.41c	176.97 ± 7.24ab	164.72 ± 9.86cd
	C2D3	89.13 ± 4.73g	132.48 ± 7.10de	133.51 ± 9.29e
	C2D4	115.83 ± 6.34de	128.74 ± 5.04de	161.12 ± 7.61cd
	C2D5	137.29 ± 5.26bc	173.10 ± 4.66b	182.72 ± 8.17b
	C3D1	112.02 ± 4.52de	130.43 ± 5.78de	178.38 ± 9.87bc
	C3D2	140.53 ± 5.78ab	179.05 ± 5.62ab	191.51 ± 10.36ab
	C3D3	103.95 ± 3.79f	144.78 ± 6.68cd	176.94 ± 7.06bc
	C3D4	135.74 ± 7.61bc	152.47 ± 8.79c	158.19 ± 10.41d
	C3D5	150.25 ± 7.40a	185.95 ± 8.57a	202.01 ± 11.09a

C1: Uniconazole; C2: Abscisic acid; C3: Zinc-amino acids chelate; D1: Seed soaking stage; D2: One leaf and one heart stage; D3: Two leaves and one heart stage; D4: 7 days before the transplanting stage; D5: regreening stage. For each treatment (C, D, or C × D), different letters in the same column indicate significant differences (P < 0.05).

### 3.2 Analysis of zinc-amino acids chelate application methods

#### 3.2.1 Rice tillering dynamics

**Figure 2:**
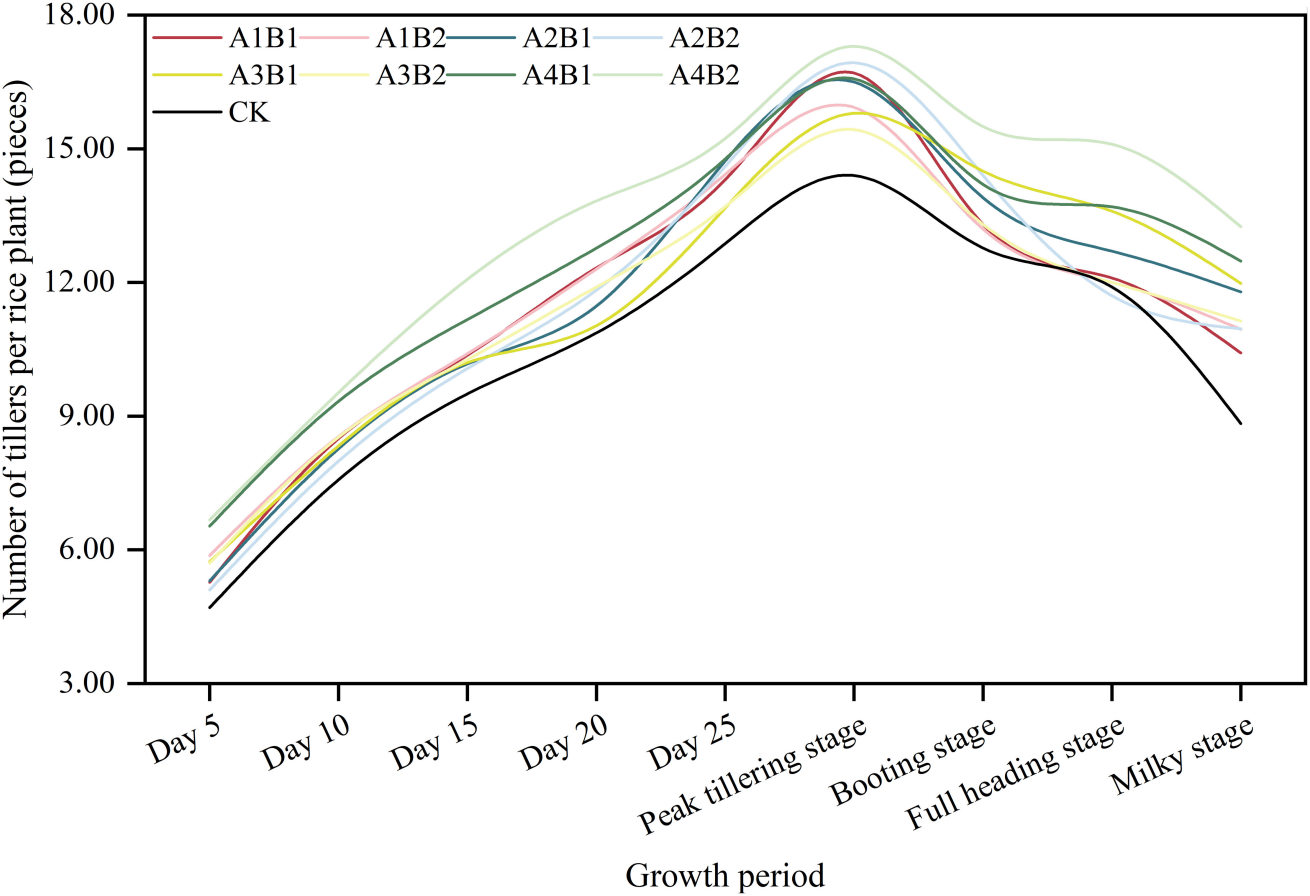
Effects of different zinc-amino acids chelate spraying amounts and times on early rice tillering dynamics. CK: control treatment; A1: 7 days before the transplanting stage; A2: Transplanting stage; A3: Regreening stage; A4: Transplanting stage and regreening stage; B1: 750 g ha^−1^; B2: 1125 g ha^−1^.

The number of tillers increased up to the peak tillering stage and then decreased (**Figure 2**). Compared with CK, the ZAC treatment increased the number of tillers. More specifically, in the peak tillering period, the number of tillers was higher for B1 and B2 (16.39 and 16.40 tillers per plant, respectively) than for CK (14.40 tillers per plant). The comparison of spraying times indicated A4 resulted in the highest number of tillers in each stage. In the peak tillering stage, the number of tillers was higher for A4 (16.93 tillers per plant) than for A2, A1, and A3 (16.72, 16.32, and 15.62 tillers per plant, respectively). In response to the combined treatments, the number of tillers in each stage was highest for A4B2, followed by A4B1, with up to 17.30 and 16.59 tillers per plant, respectively, in the peak tillering stage. Thus, spraying once with ZAC in the transplanting and regreening stages may be optimal for increasing the number of tillers, but there were no significant differences in the number of tillers between the tested dosages.

#### 3.2.2 Dry matter weight

Rice yield is influenced by the production and accumulation of specific substances. Spraying with ZAC increased the dry matter weight in the later stages (**Table 5**). In the milky and maturity stages, the dry matter weight was higher for B1 and B2 than for CK, but there was no significant difference between B1 and B2. In terms of the spraying time, there were no significant differences in the dry matter weight between the treatments at the peak tillering stage and booting stage. The dry matter weights from the full-heading stage to the maturity stage were highest for A4, followed by A3, A2, and A1. The dry matter weight for A4 was significantly higher than that for A2 and A1. For the combined treatments, there were no significant differences in the dry matter weight between treatments at the peak tillering stage and booting stage, but the differences between treatments increased significantly from the full-heading stage to the later growth stages. The dry matter weight from the full-heading stage to the maturity stage was highest for A4B2.

**Table 5:** Effects of different ZAC spraying amounts and times on the dry matter weight of early rice (g plant^-1^).

Treatment		Peak tillering stage	Booting stage	Full-heading stage	Milky stage	Maturity stage
Spraying time	A1	8.80 ± 1.41a	19.88 ± 1.17a	28.29 ± 0.81b	34.28 ± 1.55b	42.54 ± 1.62c
	A2	7.89 ± 1.27a	18.81 ± 1.27a	29.82 ± 0.69b	36.85 ± 1.78b	46.21 ± 2.02b
	A3	7.37 ± 0.83a	19.55 ± 0.94a	30.26 ± 1.13ab	41.15 ± 2.12a	48.47 ± 2.14ab
	A4	7.70 ± 1.30a	20.33 ± 2.41a	31.54 ± 1.02a	41.26 ± 2.04a	51.10 ± 2.59a
Spraying dosage	B1	7.74 ± 0.76a	20.10 ± 1.61a	29.72 ± 0.57a	38.19 ± 1.79a	46.30 ± 2.38a
	B2	8.14 ± 1.68a	19.19 ± 1.07a	29.96 ± 1.21a	38.98 ± 2.26a	47.85 ± 2.11a
Spraying time × Spraying dosage	CK	7.03 ± 0.97a	18.15 ± 0.89a	28.13 ± 1.33b	31.31 ± 1.49c	41.93 ± 2.05c
	A1B1	9.28 ± 1.43a	20.84 ± 1.68a	28.22 ± 1.06b	34.03 ± 2.16bc	42.45 ± 1.57bc
	A1B2	8.31 ± 1.55a	18.91 ± 0.72a	28.36 ± 0.89b	34.52 ± 1.73bc	42.63 ± 1.69bc
	A2B1	6.88 ± 2.19a	19.54 ± 0.80a	28.84 ± 1.12b	36.44 ± 2.01b	45.66 ± 2.31b
	A2B2	8.90 ± 1.36a	18.08 ± 1.10a	30.83 ± 1.45ab	37.25 ± 1.67b	46.75 ± 2.15b
	A3B1	7.45 ± 0.82a	20.54 ± 1.08a	30.49 ± 1.26ab	41.28 ± 1.93a	48.62 ± 1.93ab
	A3B2	7.28 ± 1.40a	18.56 ± 2.23a	30.03 ± 0.91ab	41.02 ± 2.06a	48.31 ± 2.10ab
	A4B1	7.34 ± 2.14a	19.47 ± 2.52a	31.04 ± 1.05a	41.21 ± 1.70a	48.48 ± 1.87ab
	A4B2	8.06 ± 2.25a	21.19 ± 1.58a	32.03 ± 1.83a	41.31 ± 2.12a	53.72 ± 3.04a

CK: control treatment; A1: 7 days before the transplanting stage; A2: Transplanting stage; A3: Regreening stage; A4: Transplanting stage and regreening stage; B1: 750 g ha^−1^; B2: 1125 g ha^−1^. For each treatment (A, B, or A × B), different letters in the same column indicate significant differences (P < 0.05).

#### 3.2.3 Leaf area index

Spraying with ZAC resulted in an increase in the LAI early rice, but there was no significant difference between B1 and B2 (**Table 6**). There were also no significant differences in LAI in the peak tillering stage among the spraying times. From the booting stage to the maturity stage, LAI was highest for A4, followed by A2, A3, and A1. Of these treatments, A4 and A2 resulted in a significantly higher LAI than A3 and A1. Among the combined treatments, A4B2 and A4B1 resulted in the highest LAI in each stage (the CK LAI was significantly lower).

**Table 6:** Effects of different ZAC spraying amounts and times on the early rice LAI.

Treatment		Peak tillering stage	Booting stage	Full-heading stage	Milky stage
Spraying time	A1	0.99 ± 0.16a	3.74 ± 0.22b	3.25 ± 0.18c	2.63 ± 0.17b
	A2	1.03 ± 0.10a	4.58 ± 0.29a	4.24 ± 0.31a	3.32 ± 0.28a
	A3	0.97 ± 0.08a	3.87 ± 0.40b	3.67 ± 0.22b	2.83 ± 0.20b
	A4	1.08 ± 0.13a	4.89 ± 0.37a	4.43 ± 0.39a	3.56 ± 0.24a
Spraying dosage	B1	0.99 ± 0.11a	4.21 ± 0.31a	3.80 ± 0.28a	3.02 ± 0.19a
	B2	1.04 ± 0.14a	4.33 ± 0.28a	4.00 ± 0.20a	3.15 ± 0.26a
Spraying time× Spraying dosage	CK	0.89 ± 0.09b	3.58 ± 0.15d	3.25 ± 0.15e	2.62 ± 0.16cd
	A1B1	0.97 ± 0.11ab	3.64 ± 0.12d	3.28 ± 0.12e	2.40 ± 0.13d
	A1B2	1.01 ± 0.13ab	3.83 ± 0.27cd	3.21 ± 0.11e	2.78 ± 0.16c
	A2B1	0.98 ± 0.08ab	4.71 ± 0.30ab	4.03 ± 0.16c	3.26 ± 0.18ab
	A2B2	1.08 ± 0.09a	4.43 ± 0.21b	4.44 ± 0.20ab	3.37 ± 0.26a
	A3B1	0.94 ± 0.12ab	3.72 ± 0.17cd	3.72 ± 0.14d	2.76 ± 0.22c
	A3B2	0.99 ± 0.10ab	4.02 ± 0.19c	3.61 ± 0.20d	2.90 ± 0.21bc
	A4B1	1.08 ± 0.07a	4.76 ± 0.26ab	4.15 ± 0.19bc	3.53 ± 0.33a
	A4B2	1.09 ± 0.12a	5.02 ± 0.34a	4.71 ± 0.33a	3.56 ± 0.25a

CK: control treatment; A1: 7 days before the transplanting stage; A2: Transplanting stage; A3: Regreening stage; A4: Transplanting stage and regreening stage; B1: 750 g ha^−1^; B2: 1125 g ha^−1^. For each treatment (A, B, or A × B), different letters in the same column indicate significant differences (P < 0.05).

#### 3.2.4 Yield and related components

The ZAC spray treatments increased the rice yield to some extent (**Table 7**), but the different dosages had no significant effect on yield. Compared with CK, B1 and B2 resulted in yield increases of 4.28% and 5.99%, respectively. Analyses of the yield components indicated the number of grains per panicle was slightly higher for B1 and B2 than for CK. Among the spraying times, A4 resulted in the highest rice yield, followed by A2, A3, and A1; the difference between A4 and A1 was significant. The rice yield for A4 was 9.54%, 3.67%, and 5.53% higher than that for A1, A2, and A3, respectively. In terms of the yield-related components, there were no significant differences in the number of grains per panicle, seed setting rate, and thousand-grain weight among treatments. The effective panicle number was highest for A4, followed by A2, A1, and A3; the difference between A4 and A1 was significant. For the combined treatments, the actual yield of rice was the highest in A4B2 and A4B1, and the lowest in A1B1, which were significantly higher than A1B1 by 11.40% and 9.89% respectively. The rice yields for A4B1 and A4B2 were 11.82% and 13.35% higher than that for CK, respectively. Both A4B1 and A4B2 mainly increased the effective panicle number (compared with the other treatments).

**Table 7:** Effects of different ZAC spraying amounts and times on the early rice yield and related components.

Treatment		Effective panicle (×10^4^ ha^-1^)	Grains per panicle	Seed setting rate (%)	Thousand-grain weight (g)	Theoretical Yield (t ha^-1^)	Actual yield (t ha^-1^)
Spraying time	A1	249.17 ± 10.59b	129.46 ± 5.59a	64.02 ± 3.86a	23.88 ± 1.41a	4.90 ± 0.19b	4.70 ± 0.14b
	A2	267.51 ± 13.36ab	122.23 ± 3.47a	68.27 ± 2.69a	23.40 ± 2.83a	5.20 ± 0.22ab	4.91 ± 0.19ab
	A3	254.46 ± 11.28ab	126.78 ± 4.10a	67.00 ± 4.11a	23.73 ± 1.39a	5.13 ± 0.15ab	4.88 ± 0.25ab
	A4	281.25 ± 15.12a	121.55 ± 4.16a	66.39 ± 3.36a	23.84 ± 3.07a	5.40 ± 0.28a	5.15 ± 0.31a
Spraying dosage	B1	265.17 ± 9.13a	123.25 ± 5.04a	65.69 ± 3.24a	23.79 ± 2.80a	5.08 ± 0.17a	4.87 ± 0.23a
	B2	260.92 ± 12.81a	126.76 ± 4.29a	67.15 ± 5.08a	23.63 ± 1.45a	5.23 ± 0.26a	4.95 ± 0.30a
Spraying time × Spraying dosage	CK	257.29 ± 11.14bc	116.99 ± 3.46c	66.24 ± 2.45abc	23.60 ± 2.74a	4.72 ± 0.20c	4.57 ± 0.17c
	A1B1	267.46 ± 9.52ab	124.47 ± 3.51bc	61.23 ± 2.52c	23.97 ± 1.24a	4.85 ± 0.17c	4.65 ± 0.21c
	A1B2	231.37 ± 12.09c	134.45 ± 5.32a	66.80 ± 2.69ab	23.78 ± 2.55a	4.94 ± 0.21bc	4.75 ± 0.16bc
	A2B1	261.06 ± 10.31b	119.81 ± 3.77bc	70.01 ± 3.61a	23.41 ± 2.43a	5.11 ± 0.15abc	4.87 ± 0.22abc
	A2B2	274.93 ± 13.28ab	124.64 ± 4.24bc	66.52 ± 3.19abc	23.39 ± 1.25a	5.29 ± 0.23abc	4.96 ± 0.30abc
	A3B1	260.45 ± 9.18b	127.16 ± 5.56ab	64.33 ± 2.04bc	23.88 ± 3.36a	5.06 ± 0.19abc	4.81 ± 0.24abc
	A3B2	249.68 ± 13.56bc	126.40 ± 4.26ab	69.67 ± 3.27a	23.57 ± 2.61a	5.20 ± 0.24abc	4.93 ± 0.19abc
	A4B1	272.57 ± 15.26ab	121.56 ± 5.27bc	67.18 ± 3.03ab	23.89 ± 1.77a	5.31 ± 0.26ab	5.11 ± 0.21ab
	A4B2	289.23 ± 17.35a	121.53 ± 4.69bc	65.59 ± 2.28abc	23.78 ± 1.95a	5.49 ± 0.34a	5.18 ± 0.25a

CK: control treatment; A1: 7 days before the transplanting stage; A2: Transplanting stage; A3: Regreening stage; A4: Transplanting stage and regreening stage; B1: 750 g ha^−1^; B2: 1125 g ha^−1^. For each treatment (A, B, or A × B), different letters in the same column indicate significant differences (P < 0.05).

#### 3.2.5 Rice leaf cold resistance characteristics

Compared with the CK treatment, spraying with ZAC significantly increased the rice leaf antioxidant capacity in each stage (**Table 8**). For the combined treatments, the activities of SOD, POD and CAT in leaf at each stage were highest in A4B2 and A4B1, and lowest in A1B2 and A1B1. There were no significant differences between the spray dosages. Except for CAT activity, there were significant differences in SOD and POD activities from peak tillering stage to milky stage among the spraying times (A1, A2, A3, and A4).

**Table 8:** Effects of different ZAC spraying amounts and times on the early rice antioxidant enzyme activities.

Enzyme	Treatment	7 days after the regreening stage	Peak tillering stage	Booting stage	Milky stage
SOD (U g^-1^)	CK	74.73 ± 5.57d	81.32 ± 4.93e	92.82 ± 6.83e	59.11 ± 4.34d
	A1B1	103.28 ± 7.26c	116.01 ± 6.19d	121.41 ± 10.56d	83.63 ± 8.05c
	A1B2	107.29 ± 9.28c	125.43 ± 7.55d	126.43 ± 8.10d	80.20 ± 7.59c
	A2B1	150.59 ± 12.90b	180.43 ± 10.11b	206.42 ± 10.60b	111.13 ± 8.32b
	A2B2	179.11 ± 10.26b	185.21 ± 11.13b	211.31 ± 9.49b	113.70 ± 9.46b
	A3B1	160.64 ± 9.06b	157.79 ± 9.87c	174.43 ± 10.25c	98.50 ± 10.20b
	A3B2	157.75 ± 12.39b	155.63 ± 7.41c	180.41 ± 8.21c	101.30 ± 8.81b
	A4B1	204.01 ± 14.17a	215.18 ± 18.38a	232.32 ± 13.18a	143.53 ± 14.12a
	A4B2	215.42 ± 18.42a	220.77 ± 15.79a	236.42 ± 11.64a	157.35 ± 10.44a
POD [U·(g·min)^-1^]	CK	83.32 ± 8.41c	98.32 ± 9.46e	143.73 ± 6.14e	84.68 ± 5.13e
	A1B1	150.05 ± 10.39b	160.03 ± 11.84d	188.22 ± 5.58d	109.61 ± 7.51d
	A1B2	155.78 ± 11.09b	167.68 ± 8.75d	184.58 ± 8.20d	106.72 ± 7.26d
	A2B1	166.03 ± 13.36b	204.10 ± 9.89b	229.55 ± 9.78b	145.23 ± 9.68b
	A2B2	176.25 ± 10.74b	209.60 ± 7.96b	231.94 ± 8.90b	150.03 ± 8.76b
	A3B1	171.09 ± 12.34b	184.00 ± 7.38c	208.11 ± 10.32c	122.27 ± 7.98c
	A3B2	160.17 ± 16.29b	185.91 ± 8.12c	209.88 ± 7.05c	125.67 ± 10.06c
	A4B1	208.47 ± 15.90a	226.88 ± 9.07a	250.68 ± 9.27a	168.49 ± 11.25a
	A4B2	200.69 ± 13.61a	229.21 ± 12.70a	253.99 ± 12.85a	167.34 ± 9.33a
CAT [U·(g·min)^-1^]	CK	90.64 ± 6.48d	95.63 ± 6.11d	125.63 ± 7.26c	62.39 ± 4.31e
	A1B1	112.02 ± 4.35c	128.75 ± 6.93c	189.75 ± 10.79b	78.30 ± 3.09d
	A1B2	103.82 ± 5.18c	129.52 ± 7.42c	189.52 ± 9.21b	80.22 ± 6.11d
	A2B1	127.05 ± 7.63b	146.20 ± 8.95b	196.00 ± 6.08b	108.36 ± 6.17b
	A2B2	125.69 ± 6.10b	148.67 ± 6.76b	198.67 ± 9.26b	112.10 ± 5.64b
	A3B1	133.62 ± 7.54b	146.37 ± 4.16b	196.37 ± 10.15b	87.54 ± 4.52c
	A3B2	131.42 ± 9.09b	147.70 ± 8.71b	197.70 ± 8.43b	90.61 ± 5.26c
	A4B1	153.43 ± 8.23a	163.11 ± 7.60a	240.17 ± 13.06a	127.85 ± 7.47a
	A4B2	151.69 ± 10.58a	165.27 ± 10.53a	245.09 ± 11.55a	129.23 ± 7.02a

CK: control treatment; A1: 7 days before the transplanting stage; A2: Transplanting stage; A3: Regreening stage; A4: Transplanting stage and regreening stage; B1: 750 g ha^−1^; B2: 1125 g ha^−1^. For each antioxidant enzyme (SOD, POD, and CAT), different letters in the same column indicate significant differences (P < 0.05).

## 4 Discussion

### 4.1 Screening of suitable cold-resistant agents for machine-transplanted early rice

Low temperatures seriously affect plant growth and threaten global food security. The application of foliar fertilizers can regulate various plant physiological processes and alleviate the harmful effects of adverse environmental conditions. Foliar fertilizers are quickly absorbed by plants, even at low dosages (i.e., high efficiency). Hence, they are commonly applied to increase crop quality and stress resistance. Compared with simple foliar fertilizers, compound foliar fertilizers provide plants with more diverse nutrients and plant growth-regulating substances (Niu et al., 2021). In the current study, the rice yield following the ZAC treatment was 9.22% and 7.14% higher than the rice yield resulting from the uniconazole and abscisic acid treatments, respectively. The comparison of spraying times revealed the spray application of foliar fertilizer during the regreening stage produced the highest early rice yield. Analyzing the yield related components, the ZAC treatment during the regreening stage significantly increased the number of grains per panicle and the seed setting rate of early rice, ultimately leading to the largest increase in yield.

Rice yield is mainly influenced by rice tillering, coordinated sink–source relationships, and the accumulation of dry matter. In this study, the number of tillers and dry matter weight were highest in each stage following the ZAC treatment. Moreover, the application of ZAC resulted in the highest LAI. In terms of the spraying time, the regreening stage was best for enhancing various yield-related parameters. Recent research showed that zinc fertilizers primarily improve photosynthetic activities in leaves and the absorption and utilization of nutrients (Prakash et al., 2022). Amino acids are essential nutrients for plant growth and development. It can convert inorganic substances to their organic chelated states, thereby significantly improving nutrient absorption, accumulation, and utilization rates of rice, while also promoting robust plant growth (Xie et al., 2015), ultimately enhancing the grain filling process and decreasing the number of empty grains (Navizaga et al., 2017). Therefore, compared with simple foliar fertilizers, compound foliar fertilizers are more effective for optimizing plant growth and development and increasing rice yields.

Antioxidant enzymes (i.e., CAT, SOD, and POD) are critical for balancing reactive oxygen species accumulation and removal when crops are subjected to stress (Shah and Nahakpam, 2012). They can inhibit membrane lipid peroxidation, minimize the damages to cell membranes caused by active oxygen, and delay cell aging (Xiao et al., 2011). Therefore, antioxidant enzyme activities can reflect the ability of crops to tolerate diverse stresses. Previous study showed that the application of various compounds, including paclobutrazol, abscisic acid, and uniconazole, can increase antioxidant enzyme activities and the production of secondary metabolites in rice (Wang et al., 2023). Additionally, cell membranes may be stabilized and the activities of intracellular substances may increase; these changes can increase the cold stress resistance of rice plants (Baninasab, 2009; Chen et al., 2017; Wu et al., 2023). In the present study, antioxidant enzyme activities were higher in the leaves treated with ZAC than in the leaves treated with the other cold-resistant agents. Accordingly, compared with abscisic acid and uniconazole, ZAC can more effectively maintain antioxidant enzyme activities, with beneficial effects on early rice performance under low-temperature stress conditions. Of the spraying times selected for this study, the treatments during the regreening stage resulted in the highest antioxidant enzyme activities.

Zinc is an activator and co-factor of many enzymes. During plant growth and development, CO_2_ fixation, biofilm maintenance, and auxin synthesis are all regulated by zinc-containing enzymes (Chantal et al., 2013). In plants, phytic acid and phosphoric acid combine with zinc ions to form insoluble chelates. The adsorption of zinc ions by the cell wall substantially decreases zinc transport and bioavailability in plants (Liu et al., 2023). As a better type of organic chelating agent, amino acids can convert inorganic zinc into its organic chelated state, significantly improving the zinc utilization rate in rice. In addition, amino acid foliar fertilizers are easily absorbed by crops. Notably, they can increase disease resistance and crop quality, which may be related to their positive effects on protective enzyme activities and cell stability (Kitir et al., 2019). Earlier research indicated that spraying plants with amino acid foliar fertilizers can promote leaf physiological activities and root growth and development, increase chlorophyll contents, and significantly improve the quality of vegetative tissues (Shehata et al., 2011). Hence, compared with simple foliar fertilizers, such as abscisic acid and uniconazole, amino acid-based compound foliar fertilizers contain more major nutrients, which can improve crop growth and development and increase stress resistance.

### 4.2 Zinc-amino acids chelate application methods

The application of cold-resistant agents is important for increasing early rice cold resistance and yield. However, different spraying times and dosages have diverse effects on rice cold resistance and yield. This study preliminarily demonstrated that compared with abscisic acid and uniconazole, ZAC had a better effect on early rice cold resistance and yield. Nevertheless, the ZAC dosage and spraying time may need to be further optimized for commercial rice production. In this study, compared with CK, the foliar spray application of ZAC increased the rice yield. More specifically, the actual yields after the B1 and B2 treatments increased by 4.28% and 5.99%, respectively. These results are consistent with those of earlier studies by Tian et al. (2015) and Song et al. (2008). According to the examination of specific yield-related components, the increase in the rice yield was mainly due to increases in the number of grains per panicle. There were no significant differences in rice yield between the tested dosages. Therefore, to minimize the cost of fertilizer applications, early rice plants should be treated with 750 g ha^−1^ ZAC (B1). There were significant differences in the early rice yield among ZAC spraying times. The rice yield was highest for A4, with an actual yield that was 9.54%, 3.67%, and 5.53% higher than those of A1, A2, and A3, respectively. The A4 treatment mainly increased the effective panicle number.

These results suggest that multiple applications of ZAC in the early rice growth stage may increase the final rice yield by increasing the effective panicle number. As one of the yield-related components, the effective panicle number depends on the tillering process during the vegetative growth phase, which may be related to the effects of arginine and glutamine on plant growth and development. One of the most significant characteristics of rice in the early vegetative growth stage is its strong tillering ability. Arginine is an essential amino acid for plants, but it also considerably promotes rice cell division (Itoh et al., 2005). Glutamine can effectively induce the accumulation of photosynthetic products in rice leaves, while also converting inorganic carbon to required organic carbohydrates, thereby ensuring the early nutritional needs of rice are met and inhibiting the formation of ineffective tillers (Ohashi et al., 2018).

In this study, compared with CK, the ZAC treatment positively modulated rice tillering and led to increases in LAI and the aboveground dry matter weight. Zinc is an essential element for rice growth. The application of zinc fertilizers will promote the growth and development of rice plants, improve stress resistance, increase the number of tillers, and increase the grain yield (Raza et al., 2023). In the present study, different ZAC application amounts had no significant effects on early rice yield-related characteristics. In contrast, there were differences in specific yield-related traits among the different application times, with A4 revealed as the best treatment. The tiller number, LAI, and dry matter weight at each growth stage increased most significantly. Furthermore, compared with CK, the ZAC treatment significantly increased CAT, SOD, and POD activities. There were no differences in the antioxidant enzyme activities following the B1 and B2 treatments (different dosages), but of the analyzed application times, the A4 treatment resulted in the highest antioxidant enzyme activities.

In the middle and lower reaches of the Yangtze River in China as well as in the South China region, low temperatures in spring (i.e., until the end of April) can affect early rice seedlings, which are transplanted during this period. Therefore, spraying early rice seedlings with cold-resistant agents may positively affect the subsequent plant growth and development. In this study, there were no significant differences in the early rice yield and antioxidant enzyme activities between the ZAC treatment dosages, implying dosages exceeding 750 g ha^−1^ have no additional beneficial effects. To maximize the effects of ZAC, plants should be sprayed once during the transplanting stage and the regreening stage. Spraying cold-resistant agents during the seedling stage can improve seedling quality by activating stress response mechanisms, leading to increased cold tolerance (Phutdhawong et al., 2014). Because rice seedlings have relatively small leaves, it may be difficult for the seedlings to fully absorb the cold-resistant agent. Hence, excessive or single spray applications may not be ideal. Alternatively, the application of a small amount of compound foliar fertilizer before and after the rice transplanting stage may be more effective for improving cold resistance than a single application of a relatively high fertilizer dosage, but this will need to be experimentally verified.

## 5 Conclusions

Among the three analyzed cold-resistant agents, ZAC was best for promoting rice tillering and increasing the plant LAI, dry matter weight, antioxidant enzyme activities, cold resistance, and yield. Although different amounts of ZAC had no significant effect on the early rice yield and cold resistance, there were differences among the effects of the tested spraying times. For each dosage, the early rice yield and cold resistance was highest when samples were treated with ZAC once during the transplanting stage and the regreening stage. Therefore, spraying early rice plants with 750 g ha^−1^ ZAC once during the transplanting stage and the regreening stage may be the ideal treatment for optimizing cold resistance and yield.

## Data Availability

The original contributions presented in the study are included in the article/**Supplementary Material**. Further inquiries can be directed to the corresponding authors.
