# Supplementary figures and images for: Effects of different cold-resistant agents and application methods on yield and cold-resistance of machine-transplanted early rice
Source: Front Plant Sci. 2024 Oct 2;15:1422374. doi: 10.3389/fpls.2024.1422374 (PMC11480013; doi:10.3389/fpls.2024.1422374)

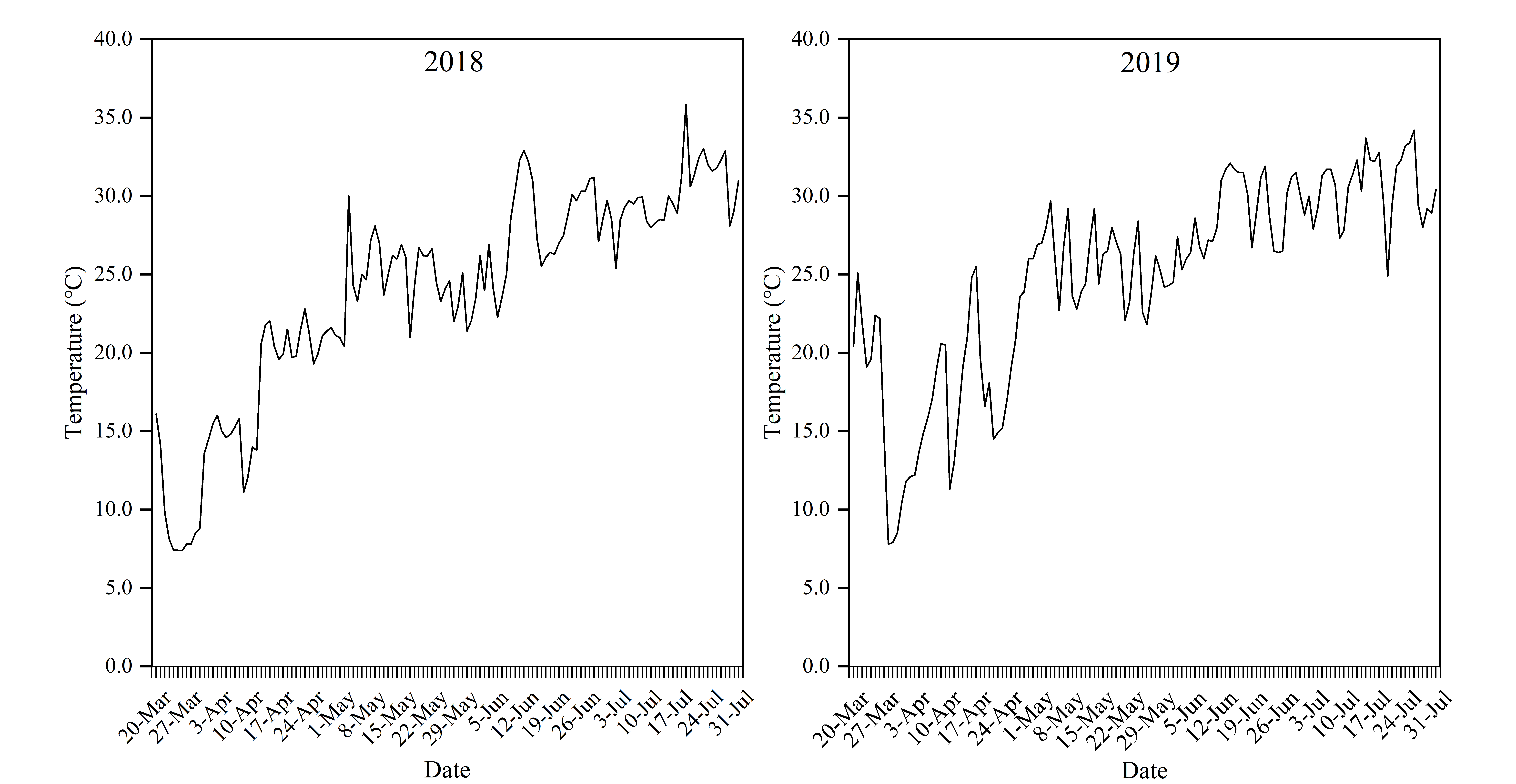


**Fig. S1.** Average daily temperatures from March to July in 2018 and 2019

Supplement: Supplementary file 1 [file DataSheet1.docx]
